# Hemodynamic and genetic analysis in children with idiopathic, heritable, and congenital heart disease associated pulmonary arterial hypertension

**DOI:** 10.1186/1465-9921-14-3

**Published:** 2013-01-09

**Authors:** Nicole Pfarr, Christine Fischer, Nicola Ehlken, Tabea Becker-Grünig, Vanesa López-González, Matthias Gorenflo, Alfred Hager, Katrin Hinderhofer, Oliver Miera, Christian Nagel, Dietmar Schranz, Ekkehard Grünig

**Affiliations:** 1 Institute of Human Genetics, University of Heidelberg, Heidelberg, Germany; 2 Centre of Pulmonary Hypertension Thoraxclinic, University Hospital Heidelberg, Amalienstrasse 5, 69126, Heidelberg, Germany; 3 Unidad de Genética Médica, Servicio de Pediatría, Hospital Universitario Virgen de La Arrixaca, Murcia, Spain; 4 Department of Paediatric Cardiology, University of Heidelberg, Heidelberg, Germany; 5 Department of Pediatric Cardiology and Congenital Heart Disease, Deutsches Herzzentrum München, Technische Universität München, Munich, Germany; 6 German Heart Center, Berlin, Germany; 7 Department of Paediatric Cardiology, University of Giessen, Giessen, Germany

**Keywords:** Pulmonary hypertension, Congenital heart disease, Genetics, Children, Bone morphogenetic protein receptor 2

## Abstract

**Background:**

Aim of this prospective study was to compare clinical and genetic findings in children with idiopathic or heritable pulmonary arterial hypertension (I/HPAH) with children affected with congenital heart defects associated PAH (CHD-APAH).

**Methods:**

Prospectively included were 40 consecutive children with invasively diagnosed I/HPAH or CHD-APAH and 117 relatives. Assessment of family members, pedigree analysis and systematic screening for mutations in TGFß genes were performed.

**Results:**

Five mutations in the bone morphogenetic protein type II receptor (BMPR2) gene, 2 Activin A receptor type II-like kinase-1 (ACVRL1) mutations and one Endoglin (ENG) mutation were found in the 29 I/HPAH children. Two mutations in BMPR2 and one mutation in ACVRL1 and ENG, respectively, are described for the first time. In the 11 children with CHD-APAH one BMPR2 gene mutation and one Endoglin gene mutation were found. Clinical assessment of relatives revealed familial aggregation of the disease in 6 children with PAH (HPAH) and one CHD-APAH patient. Patients with mutations had a significantly lower PVR.

**Conclusion:**

Mutations in different TGFß genes occurred in 8/29 (27.6%) I/HPAH patients and in 2/11 (18.2%) CHD-APAH patients and may influence the clinical status of the disease. Therefore, genetic analysis in children with PAH, especially in those with I/HPAH, may be of clinical relevance and shows the complexity of the genetic background.

## Introduction

Pulmonary arterial hypertension (PAH) is a very rare disease in childhood and can occur idiopathic (IPAH), heritable (HPAH) or associated with various diseases (APAH) including congenital heart defect (CHD-APAH) [1]. As soon as familial aggregation or a genetic defect has been identified in IPAH patients they will be classified as HPAH. The true incidence of IPAH, HPAH or CHD-APAH has not yet been established [2]. However, estimates are around 2.2 new cases per million per year in the general population [3] suggesting that a few hundred children are affected in Germany. Children with I/HPAH can have similar clinical presentation as CHD-APAH patients with identical histological findings including plexiform lesions [4].

I/HPAH and CHD-APAH are the most frequent cause for PAH in children [5]. The classification of paediatric patients into these groups is difficult in some cases because they often present with multiple symptoms and clinical manifestations [6]. Furthermore, PAH in infancy is frequently associated with genetic syndromes [6]. In 13% of children with pulmonary hypertension chromosomal anomalies such as Trisomy 21 are reported [7].

It is well known that genetic factors predispose some individuals to develop PAH [2]. Especially heterozygous mutations in the bone morphogenetic protein receptor type II (BMPR2) gene on chromosome 2q33 were found in > 70-80% of adult PAH patients with familial history of disease [8-12]. In sporadic IPAH patients without reported familial history of the disease and in children, BMPR2 mutations were identified much less frequently (in 10-40% of cases [9,13-15]). BMPR2 mutations are even less common in patients with CHD-APAH. Therefore, an additional genetic background has been postulated [16].

Previous studies suggested that adult BMPR2 mutation carriers are younger at diagnosis with a more severe hemodynamic compromise [12,17]. Patients carrying an activin A receptor type II-like 1 (*ACVRL1*) mutation have been characterized to be even younger at diagnosis and death, compared to patients with BMPR2 mutations or without mutations [18]. Similar results have been seen in a mixed cohort with 15 adult and 8 paediatric mutation carriers [15]. There are no correlation studies yet comparing genetic and clinical findings especially in children with PAH. Two recent studies have identified mutations in the ACVRL1 (also known as ALK1) gene in several paediatric I/HPAH cases [14,19]. In a child with PAH followed by the development of heritable hemorrhagic telangiectasia (HHT) a mutation in the Endoglin (ENG) gene has been found [20]. Very recently two mutations in the SMAD9 gene were reported in children diagnosed with HPAH [21,22]. Thus, PAH in infancy seems to have a heterogeneous genetic background. Until now, only a few studies investigated the prevalence of mutations in the genes participating in the TGFß signalling pathway in children systematically. They consisted of small numbers of paediatric PAH patients and predominantly the BMPR2 gene was analysed. The aim of this study was to perform a broad and systematic screening for mutations in BMPR2, ACVRL1, ENG, SMAD1, SMAD5 and SMAD9 genes and to compare clinical and genetic findings in a German cohort of children affected with I/HPAH and CHD-APAH.

## Methods

### Study population

Forty unrelated children under the age of 14 years participated in this study. The diagnoses I/HPAH and CHD-APAH were established at the participating PH centers according to current guidelines [1,23]. In all patients a detailed family history was obtained and a pedigree was constructed. Relatives of index patients underwent detailed clinical examination as described previously [16,24]. All index patients underwent a detailed clinical work up including right heart catheterization and acquisition of age at onset and age at diagnosis. Their parents gave written informed consent for this study, which was approved by the Ethics Committee of the University of Heidelberg.

### Molecular genetic analysis

Genomic DNA was extracted from peripheral blood leukocytes of patients and relatives. The entire coding regions and the exon/intron boundaries of the BMPR2, ACVRL1, ENG, SMAD1, SMAD5 and SMAD9 genes were PCR amplified followed by denaturing high-performance liquid chromatography (DHPLC) as previously described [9] and/or direct sequenced using BigDye Terminator Kit v1.1 and the ABI 3100 genetic analyzer (Applied Biosystems, Foster City, CA). In mutation carriers all first degree relatives, if available, were investigated for the mutation identified in the index patient. Primers and amplification procedures are available upon request. Pathogenicity of identified sequence alterations was assessed by use of the in silico prediction programs “PolyPhen” [25], and “MutationTaster” [26], “SIFT” [27], and Alamut v2.2 (Interactive Biosoftware, [28]). For detection of whole exonic deletions /duplications the Multiplex Ligation-dependant Probe Amplification (MLPA) technique was applied according to the manufacturer’s instructions (MRC-Holland, Amsterdam).

### Statistical analysis

Hemodynamic values are given as mean values ± standard deviations and 95% confidence intervals. We compared I/HPAH with CHD-APAH and mutation carriers vs. non carriers, respectively, with respect to baseline characteristics and hemodynamic parameters by two-tailed students t-test and Chi-square tests using SPSS 20 (IBM SPSS Statistics V20 IBM Corporation). P values below 0.05 were considered statistically significant.

## Results

### Study population

**Figure 1:**
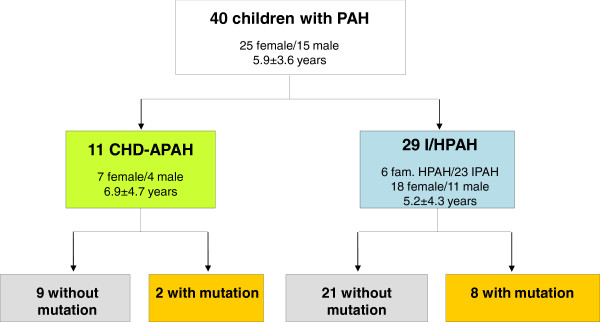
**Genetic disposition of the study population.** The figure shows the proportion of mutation carriers and non carriers in the study population, female to male proportion and the mean age at diagnosis. I/HPAH = idiopathic/hereditary pulmonary arterial hypertension, CHD-APAH = congenital heart disease associated pulmonary arterial hypertension. One BMPR2 variant has been classified as mutation but could represent a mutation or a non-deleterious polymorphism.

**Figure 2:**
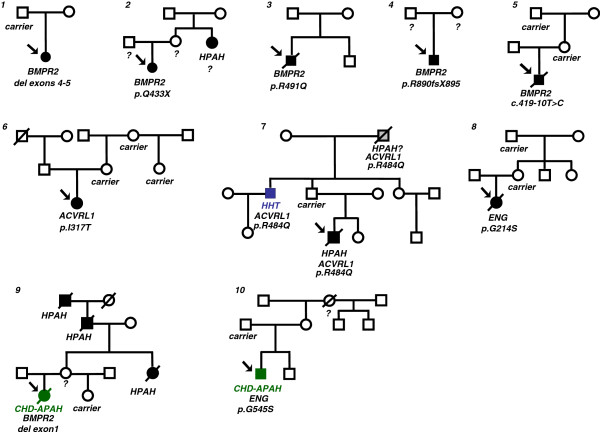
**Pedigrees of HPAH**/**CHD**-**APAH patients with identified mutations.** The figure represents pedigree trees of children with mutations. The index patient of each family is marked by an arrow. All family members with manifest PAH are shown in black. Family members in whom the diagnosis HPAH is questionable are shown in grey. Healthy family members have open symbols and those who were heterozygous for the identified mutation are referred to as “carrier”. Not tested family members are indicated by question mark. Pedigrees 1 to 8 represent families of I/HPAH patients and pedigrees 8 and 9 families of CHD-APAH patients. The numbering of the pedigrees corresponds to the appearance of the patients in Table 1. Mutations in the BMPR2 gene in I/HPAH patients were found in families 1 to 4. The BMPR2 sequence variant in family 5 which we classified as mutation could represent a mutation or a non-deleterious polymorphism. ACVRL1 mutations were present in families 6 and 7, and the ENG mutation in family 8. In CHD-APAH patients mutations were identified in BMPR2 (family 9) and ENG (family 10).

Between January 2008 and May 2012 40 children with PAH and 117 relatives of 20 index patients participated in the study. Due to pedigree analysis and clinical work up 29 children were classified as I/HPAH and 11 as CHD-APAH patients (10 atrial septal defect = ASD II, one ventricular septal defect = VSD, Figure 1). By pedigree analysis and clinical assessment of relatives in 6 initially as IPAH classified patients and in one patient with CHD-APAH familial aggregation of the disease was observed with at least one further affected family member (diagnosed by right heart catheterization) (Figure 2). One HPAH (B7944, Figure 2) and two IPAH patients (K6735 and K17353, pedigrees not shown), respectively belong to consanguineous families. In 23 of the 29 IPAH and in 10/11 CHD-APAH patients pedigree analysis and assessment of relatives (n = 69) revealed no familial aggregation of the disease. One of the IPAH cases had a suspected Russell-Silver syndrome, a clinically heterogeneous condition characterized by severe intrauterine growth retardation, poor postnatal growth, craniofacial features and a variety of minor malformations.

### Identification of mutations

In 10 of the analyzed 40 children a mutation could be identified: 8 in I/HPAH, 2 in CHD-APAH. Two mutations were detected in patients with familial aggregation of the disease (1 HPAH, 1 CHD-APAH), and 8 in clinically sporadic IPAH cases (Table 1, Figure 2). Seven of the identified mutations have been previously described (4 in BMPR2, 1 in ACVRL1, and two in ENG). We identified three so far not described sequence variants: two in the BMPR2 gene (c.419-10 T > C and p.R890GfsX6) and one in the ACVRL1 gene (p.I317T, Table 1, Figure 2). Their functional relevance was assessed using the computer prediction programs. The frame shift mutation p.R890fsX6 was clearly classified as mutation since it leads to premature insertion of a stop codon. The amino acid Ile on position 317 of the ACVRL1 protein is highly conserved and therefore the identified variant p.I317T is classified as mutation. The third novel variant is a potential splice site mutation c.419-10 T > C. We could not perform analysis of mRNA on the functional level since the child already died. The in silico prediction analysis did not reveal definitive results; therefore, this variant could be a mutation or a non-deleterious polymorphism. For the summary statistics it has been counted as BMPR2 mutation.

**Table 1:** Clinical and genetic characteristics of mutation carriers

	**Patient**	**Gender**	**Age**	**Clinical classification**	**Positive family history**	**Gene**	**Type of mutation**	**Gene region**	**Mutation**	**FM clinically assessed**	**Affected FM**	**Unaffected FM carrying mutation**
**I**/**HPAH**												
**1**	**A8317**	f	7	IPAH	no	BMPR2	deletion	Exons 4-5	c.419-?_621 + ?del	2	-	1
**2**	**A15678**	f	12	IPAH	?	BMPR2	nonsense	Exon 10	c.1297C > T (p.Q433X)	-	1 (n. a.)	n. a.
**3**	**A8205**	m	5	IPAH	no	BMPR2	missense (de novo)	Exon 11	c.1472 G > A (p.R491Q)	3	-	-
**4**	**A16735**	m	12	IPAH	?	BMPR2	frameshift	Exon 12	c.2668DelA (p.R890GfsX6) *	-	? (adopted)	n. a.
**5**	**A8643**	m	5.5	IPAH; syncope	no	BMPR2	Splicing defect?	Intron 3	c.419-10 T > C *^#^	3	-	2
**6**	**A3385**	f	3	IPAH; syncope	no	ACVRL1	missense	Exon 7	c.950 T > C (p.I317T) *	7	-	3
**7**	**A6620**	m	3.5	HPAH	yes (HHT + HPAH)	ACVRL1	missense	Exon 10	c.1451 G > A (p.R484Q)	6	2 (one HHT)	1
**8**	**A15836**	f	1.5	IPAH	no	ENG	missense	Exon 5	c.640 G > A (p.G214S)	4	-	2
**CHD**-**APAH**												
**9**	**C6710**	f	2	CHD-APAH (VSD); Fetal alcohol syndrome	Yes (HPAH)	BMPR2	deletion	Exon 1	c.1-?_76 + ?del	1	3 (all †, not tested)	1
**10**	**C3783**	m	13	CHD-APAH (ASDII); Congenital hip dislocation	no	ENG	missense	Exon 12	c.1633 G > A (p.G545S)	6	-	1

*marked mutations are described for the first time in this study; ? = unknown, n. a. = not available; *FM* family member; *ASD* atrial septal defect; *VSD* Ventricular septal defect; HHT hereditary hemorrhagic telangiectasia; † deceased, # unclassified: mutation or non-deleterious polymorphism.

The two mutations identified in ENG (1 in CHD-APAH and one in IPAH) were previously described in patients with HHT [29,30]. Both, patients and their mutation positive but unaffected family members in our cohort did not show any sign of HHT. In five of I/HPAH and in two CHD-APAH patients the identified mutations were also identified in asymptomatic family members (in 6 parents, 3 grandparents, 1 aunt and 1 sister). No mutations were identified in any of the analysed SMAD genes.

Table 1 lists all identified mutations, the type of alteration, their location in the gene, gender, the age at diagnosis, and clinical classification. New identified mutations of the investigated genes are indicated by asterisks. Common and rare polymorphisms without disease relevance have also been detected in all investigated genes (data not shown).

### Distribution and frequency of mutations

**Figure 3:**
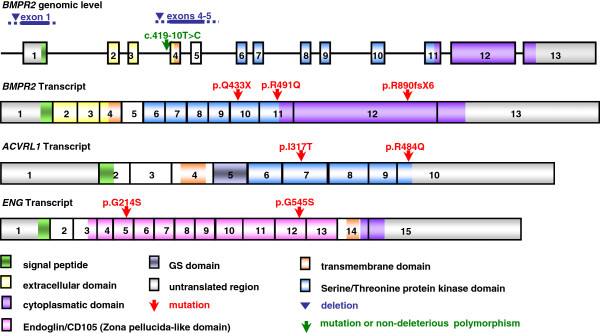
**Location of the identified mutations in the genes BMPR2, ACVRL1, and ENG.** The figure shows the location of all identified mutations in the investigated genes. Larger deletions are shown as line above the genomic gene region, point mutations (nonsense and missense), splice site mutations, frameshift mutations and small deletions are marked as arrows. The green arrow denotes the BMPR2 sequence variant which was classified as mutation but could represent a mutation or non-deleterious polymorphism. Boxes represent exons, and the colours of the boxes represent the different domains.

The 5 BMPR2 mutations identified in the study population were: 2 point mutations (1 nonsense mutation and 1 missense mutation), 1 frameshift mutation (small deletion) and 2 large deletions, and 1 potential splice site mutation (at the splice acceptor site), The nonsense, frameshift mutation and deletion of exons 4 to 5 resulted in a premature termination of the protein. Both ACVRL1 and ENG mutations were missense mutations. The BMPR2 mutations were distributed throughout the gene whereas the ACVRL1 mutations were found only in the serine/threonine protein kinase domain and the two ENG mutations were located in the endoglin/CD105 domain. The distribution of the mutations across the genes is shown in Figure 3.

### Clinical and hemodynamic characteristics

The mean age at diagnosis of all 40 investigated children was 6.0 ± 3.6 years. 25 children were females and 15 males reflecting a female to male ratio of 1.7:1 (Figure 1). Children carrying a mutation (Table 1, Figure 2) had a significantly lower pulmonary vascular resistance (PVR) than patients without mutation (Table 2). Further clinical parameters at diagnosis listed in Table 2 did not significantly differ between mutation carriers and non carriers. HPAH patients carrying a BMPR2 mutation were older than those carrying an ACVRL1 or the patient with the ENG mutation. Gender distribution ratios (female : male) were congruent in I/HPAH and CHD-APAH (1.9:1 vs. 1.8:1, p = 0.613) and also mean age at diagnosis (5.2 ± 4.3 years vs. 6.9 ± 4.7 years, p = 0.352). Both groups did not significantly differ in any of the analysed hemodynamic parameters (Table 2). Mean age at onset of first symptoms did not significantly differ between groups.

**Table 2:** Clinical characteristics at diagnosis

**Baseline parameters**		**I/HPAH**		**CHD-APAH**			**Mutation carriers**		**Non carriers**	
**n**		**29**		**11**			**10**		**30**	
**Female/male ratio**		**18/11**		**7/4**			**5/5**		**20/10**	
**Baseline and hemodynamic parameters**										
	**p-value**	**Mean ± SD**	**95% CI for the mean**	**Mean ± SD**	**95%CI for the mean**	**p-value**	**Mean ± STD**	**95%CI for the mean**	**Mean ± STD**	**95%CI for the mean**
**Age of onset at first symptoms** (**years**)	0.974	4.4 ± 3.9	3.0-5.9	4.5 ± 3.9	2.1-6.8	0.419	5.3 ± 4.1	2.8-7.8	4.1 ± 3.8	2.8-5.5
**Age at diagnosis by RHC** (**years**)	0.352	5,2 ± 4.3	3.1-7.3	6.9 ± 4.7	4.0-9.8	0.503	7.1 ± 4.1	3.5-10.7	5.6 ± 4.5	4.7-7.5
**Heart rate per minute**	0.744	98 ± 35	79-118	94 ± 15	84-105	0.776	93.4 ± 21.3	74.7-112.1	97.8 ± 31.3	82.4-113.1
**Oxygen Saturation** (%)	0.140	95.0 ± 6.5	91.6-98.4	81.8 ± 31.8	61.0-102.5	0.448	97.3 ± 2.2	95.1-99.4	88.3 ± 22.7	78.1-98.5
**PASP** (**mm Hg**)	0.799	87.3 ± 49.4	61.4-113.2	82.1 ± 23.8	65.5-99.7	0.275	64.5 ± 5.7	58.9-70.1	90.5 ± 45.4	69.0-112.1
**PADP** (**mm Hg**)	0.939	38.9 ± 26.1	24.7-53.1	39.8 ± 16.1	26.9-52.8	0.169	25.0 ± 11.1	14.1-35.9	43.0 ± 24.0	30.9-55.2
**PAMP** (**mm Hg**)	0.864	58.0 ± 33.0	40.0-76.0	55.5 ± 16.3	42.4-68.6	0.298	43.6 ± 6.6	37.3-50.2	60.8 ± 31.0	45.1-76.5
**PCWP** (**mm Hg**)	0.691	6.9 ± 3.3	5.1-8.8	6.2 ± 3.5	3.1-9.3	0.691	6.0 ± 4.4	1.1-10.9	6.9 ± 3.1	5.2-8.5
**SASP** (**mm Hg**)	0.998	93.7 ± 14.1	86.3-101.1	93.7 ± 13.0	85.7-101.8	0.871	94.6 ± 8.7	87.0-102.2	93.5 ± 14.5	87.0-100.0
**SADP** (**mm Hg**)	0.853	52.1 ± 16.2	43.6-60.6	53.1 ± 6.8	48.7-57.3	0.295	58.0 ± 5.4	53.2-62.8	51.1 ± 14.0	44.8-57.4
**Cardiac Index** (**L**/**min**/**m**^**2**^)	0.563	3.3 ± 1.1	2.6-3.9	3.7 ± 0.8	2.8-4.5	0.644	3.6 ± 1.1	2.6-4.7	3.3 ± 1.0	2.7-3.9
**PVRI** (Wood U*m^**2**^)	0.461	18.6 ± 16.1	9.8-27.4	12.9 ± 6.2	7.5-18.3	0.179	8.5 ± 2.5	6.1-10.9	19.4 ± 15.2	11.5-27.4
**PVR** (dyn*s*cm^−**5**^)	0.804	2012.2 ± 1788.8	1039.8-2984.5	1795.4 ± 1038.8	884.9-2705.9	0.014*	927.0 ± 284.4	1039.8-2984.5	2244.8 ± 1692.5	884.9-2705.9

Values are mean ± Standard Deviation = SD; *RHC* right heart catheterization, *CI* confidence interval, *PASP* pulmonary arterial systolic pressure, *PAMP* mean pulmonary arterial pressure, *PADP* diastolic pulmonary arterial pressure; *PCWP* pulmonary capillary wedge pressure; *SASP* Systemic systolic arterial pressure; *SADP* systemic diastolic arterial pressure, *PVRI* pulmonary vascular resistance index.

* p < 0.05.

## Discussion

The results of the study indicate that the proportion of genetic alterations in children with various forms of PAH is higher than previously thought and is related to several different genes of the TGFß pathway. Mutations of the BMPR2, ACVRL1, and ENG gene, respectively, occurred in 8 out of 29 (27.5%) of I/HPAH patients and even in 2 of 11 patients (18.2%) with CHD-APAH. Children carrying a mutation had a significantly lower PVR than non carriers. Two BMPR2 and one ACVRL1 mutation detected in this study have not been described before. Genetic analysis in children with various forms of PAH may be of clinical relevance and shows the complexity of the genetic background in childhood.

### Prevalence of gene alterations in children with I/HPAH

The prevalence of the here identified mutations in 27.5% of I/HPAH patients in the BMPR2, ACVRL1 and ENG genes is similar as previously reported. In 22% of 18 I/HPAH-children Harrison et al. found mutations in these 3 genes [14]. Rosenzweig et al. found in 10% of patients BMPR2 mutations [15]. In contrast to these 3 genes sequence alterations of the SMAD genes (SMAD1, SMAD5 and SMAD9) seem to be a rare event in adult or paediatric PAH patients (<2%) with unclear functional significance [22]. In this study we identified no mutation in any of the SMAD genes, but 3 rare polymorphisms in SMAD9 in 3 I/HPAH patients.

### Genetic findings in children with CHD-APAH and classification

The findings of this study (mutations in 18.2%) confirm that gene defects play also a role in CHD-APAH-patients. However, BMPR2 mutations have been a rare finding in CHD-APAH and occurred in only 6% of cases of a mixed cohort of adults and children [31]. In our CHD-APAH-patient we found a deletion of the first exon of the BMPR2 gene which has been previously observed only in I/HPAH [32,33]. Although such deletions are predicted to cause an altered translation, their penetrance is variable and clinically unaffected mutation carriers have also been reported [33] and have been seen in the sister of our index patient. HPAH and CHD-APAH are simultaneously present in this family. Thus, the classification in HPAH and CHD-APAH according to the guidelines [1,34] seems to be difficult in this case since both forms can occur in one patient.

### Mutations of ACVRL1/ENG and HHT in childhood

This study shows that ACVRL1 and ENG mutations can cause the development of severe PAH in childhood, without any symptoms or familial history of HHT. Wide intrafamilial phenotypic variations were previously observed in carriers of a mutation at position 484 [35]. Some of the mutation carriers in these families showed symptoms of HHT, others of pulmonary hypertension and one a combination of both phenotypes [35], as was seen in our family with altered amino acid at this position (p.R484Q). The novel mutation p.I317T identified in patient A3385 was also present in three unaffected family members. Neither the index patient nor the mutation carriers in this family showed any clinical signs of HHT and occurrence of HHT was also not reported in the family history.

In our cohort we have detected two missense mutations in the ENG gene: one in a patient with IPAH (A15836, p.G214S), and one in a patient with CHD-APAH (D3783). ENG mutations are quite common in HHT patients and both mutations have previously been described in patients with HHT only [29-31]. In our patients neither the index patient nor any of the mutation positive family members showed any clinical signs of HHT.

The ENG mutations identified in a CHD-APAH patient and in an IPAH patient without phenotypic appearance of HHT sheds new light on the variability of the phenotype caused by defects in TGFß pathway genes. Both mutations lead to an exchange from a highly conserved glycine residue to a serine residue but are located on different sites of the protein: p.G214S at the N-terminal and p.G545S at the C-terminal part of the endoglin/CD105 domain. They are both considered to be damaging predicted by PolyPhen [25].

Beside the here identified genetic defects in children with I/HPAH and CHD-APAH there are most likely further not yet identified genetic factors, in some families possibly inherited in a recessive mode of inheritance as previously suggested [16].

### Clinical relevance of genetic alterations

We compared hemodynamic parameters between the 10 mutation carriers and 30 non carriers. In our study, patients with mutation had a significantly lower PVR than patients without mutation. Due to the small number of patients we cannot exclude that this difference could have been accidentally occurred particularly with respect to the not significantly different PVRI. Rosenzweig et al. [15] found no significant differences in PVRI between mutation carriers and non carriers as well. These comparisons are anyhow difficult with respect to the genetic heterogeneity in this population.

### I/HPAH vs. CHD-APAH

In this study we did not find significant differences in hemodynamic parameters or age at onset between I/HPAH and CHD-APAH patients. This is in contrast to data from Barst et al. [36] and Hill et al. [37] who both found a significantly higher PVRI and an older age at onset of the disease in I/HPAH patients. Again this might be due to the relatively small numbers of patients. Further studies with larger cohorts are needed to investigate the differences between I/HPAH and CHD-APAH.

In conclusion, mutations in different TGFß genes occurred in 8 out of 29 I/HPAH patients and 2 out of 11 CHD-APAH-patients and may influence the clinical status of the disease. Therefore, genetic analysis in children with PAH may be of clinical relevance and shows the complexity of the genetic background.

## Competing interests

The authors declare that they have no competing interests.

## Authors’ contributions

NP and KH carried out the molecular genetic studies; NP drafted the manuscript and evaluated the molecular genetic data. CF performed the statistical analysis and drafted the manuscript. NE, TBG, CN, VLG, MG, OM, AG, DS and EG treated the patients and collected data. EG conceived of the study, and participated in its design and coordination and drafted the manuscript. All authors read and approved the final manuscript.
